# Chlorogenic Acid: Characteristics, Neuroprotective Effects, and Potential Mechanisms

**DOI:** 10.2174/011570159X459115260204170117

**Published:** 2026-04-09

**Authors:** Jingjing Zeng, Peiru Wang, Zhongyu Fan, Guoqing Wang, Ji Zhou, Feng Zhang

**Affiliations:** 1 Key Laboratory of Basic Pharmacology of Ministry of Education and Joint International Research Laboratory of Ethnomedicine of Ministry of Education and Key Laboratory of Basic Pharmacology of Guizhou Province and Laboratory Animal Center, Zunyi Medical University, Zunyi, Guizhou, China

**Keywords:** Chlorogenic acid, herbs, neuroprotective effects, neuropsychiatric disorders, neuroprotection, polyphenol

## Abstract

In this review, we explore recent evidence connecting chlorogenic acid (CGA) to neuropsychiatric disorders and critically discuss the biological mechanisms underlying these effects. CGA is a natural polyphenol that usually exists in fruits and vegetables. CGA has long been recommended for its broad pharmacological activities. Increasing evidence from animal studies has revealed that dietary CGA supplementation may confer protective effects on the nervous system. Here, we summarize multiple findings on CGA in animal models of neuropsychiatric disorders, including Parkinson’s disease, Alzheimer’s disease, stroke, depression, epilepsy, and other neuropsychiatric disorders. We place equal emphasis on chemical features, natural sources, bioavailability, and pharmacological properties of CGA; all of these can have a critical impact on CGA’s intervention. Current experimental evidences suggest that the neuroprotective effects of CGA are driven by the convergence of several processes, including suppression of neuroinflammation, attenuation of oxidative stress, and context-dependent effects on synaptic and cellular homeostasis. In some models, CGA has also been associated with changes in autophagic activity and reduced accumulation of misfolded or aggregated proteins. Despite these advances, the field still lacks a coherent molecular framework that links CGA exposure to specific neural outcomes. Therefore, resolving this gap will be essential for the clinical application of CGA.

## 1 INTRODUCTION

 Neuropsychiatric disorders, such as Parkinson’s disease (PD), Alzheimer’s disease (AD), stroke, depression, and epilepsy, can impose a growing global disease burden [1]. Despite numerous studies, current treatments still remain limited and usually fail to prevent disease progression. At the same time, growing concern regarding the negative impacts of chemical drugs further stimulated public interest in natural compounds as alternatives [2]. Growing experimental studies suggest that naturally derived polyphenols exert measurable neuroprotective effects, exhibiting their potential in the progression of neuropsychiatric disorders [3]. In terms of mechanism, these polyphenols appear to influence multiple signaling pathways in neuroinflammation, oxidative stress, and synaptic dysfunction. Among these polyphenols, chlorogenic acid (CGA) has attracted considerable attention due to its wide dietary availability and biological activity. Generally speaking, CGA refers to a group of hydroxycinnamic acid esters, including caffeoyl-, coumaroyl-, feruloyl-, and dicaffeoyl-substituted quinic acids [4].

As one of the predominant polyphenolic compounds in the human diet, CGA can be easily found in various plant-derived foods. Notably, coffee serves as the main source of CGA in the daily diet [5, 6]. Although CGA has been associated with diverse biological effects, its precise role in neuropsychiatric disorders remains to be fully elucidated. Additionally, each subgroup of CGA has various isomeric forms, with different coffee extracts containing varying CGA isomers. Prevalent forms in coffee are 3-caffeoylquinic acid(3-CQA), cryptochlorogenic acid (4-CQA), and neochlorogenic acid (5-CQA) [7]. Many experimental studies have revealed that CGA exhibits remarkable neuroprotective effects, supporting its potential as a therapeutic candidate in neuroprotective properties [8].

CGA is widely utilized in the food, chemical, pharmaceutical, and health care industries. Previous studies have suggested that CGA has protective potential in neuropsychiatric disorders. Hitherto, almost no review systematically elaborates on CGA’s neuroprotective effects in animal models. And a recent review articulated the relationship between CGA and neuropsychiatric disorders involving 5-CQA in 2017 [9]. In order to further update the research on the neuroprotective effect of CGA, this review concluded the characteristics of CGA and its neuroprotective effects based on studies using animal models published between 2016 and 2025. It is worth mentioning that in 1976, the International Union of Pure and Applied Chemistry (IUPAC) reversed the numbering order of atoms on the quinic acid ring and proposed that CGA should be renamed as 5-CQA. However, many chemical suppliers and researchers continue to use the pre-IUPAC nomenclature, referring to CGA as 3-CQA. This is likely since when IUPAC introduced the new numbering system for cyclitols in 1976, the name for CGA had already been established as 3-caffeoylquinic acid, and the pure form of neochlorogenic acid wasn’t yet available [8]. To avoid this confusion, readers should pay attention that in this review, we have adhered to the conventional nomenclature for CGA, which is widely used in most literature and web pages.

## 2 CHARACTERISTICS OF CGAS

CGAs consist of a group of polyphenols and have been extensively studied for their biological potential. CGAs are formed through the reaction between quinic acid and trans-cinnamic acids. This reaction results in multiple structural molecular forms of CGAs. These variations, together with differences in dietary origin, bioavailability, and pharmacological effects, play a crucial role in the wide range of biological effects.

Fig. (**1**) presents the key features of CGAs, from their chemical structures to their major food sources, bioavailability, and pharmacological actions (Fig. **1**). In Panel A, we articulate the generation process of CGA and its isomers. To be specific, the reaction between caffeic acid and quinic acid generates several isomers of CGAs, among which 3-CQA, 4-CQA, and 5-CQA are the most prevalent forms. These isomers differ in the position of the caffeoyl group on the quinic acid ring, and these structural differences can influence the stability, solubility, and metabolic transformation of CGA. Panel B summarizes major dietary sources of CGA and outlines its metabolic digestive process. During digestion, CGA is converted into its isomers, including 3-CQA, 4-CQA, and 5-CQA. Approximately one-third of CGA is absorbed in the small intestine, while the majority passes into the large intestine, where it further experiences transformation by the gut microbiota. Panel C highlights the crucial pharmacological effects of CGA, including actions related to oxidative balance, inflammatory signaling, glucose and lipid metabolism, microbial growth, and tumor-associated pathways. Taken together, the available evidence indicates that both chemical structure and its metabolism are central in determining how CGA exerts its neuroprotective effects on neural and behavioral outcomes, particularly in neuropsychiatric disorders.

### 2.1 Chemistry

CGAs comprise a group of quinic acid esters, and they are usually formed through esterification reactions between quinic acid and trans-cinnamic acid derivatives [10]. The most common form of CGA is generated by the linkage of one caffeic acid molecule to one quinic acid molecule, producing a compound with the molecular formula C_16_H_18_O_9_.

From a chemical perspective, CGA possesses multiple reactive functional groups, including catechol, ester, carboxyl, and hydroxyl groups, together determining its unique features. Importantly, different positions at which the caffeoyl group is esterified on the quinic acid ring lead to a series of structural isomers, such as 3-CQA, 4-CQA, 5-CQA, 1,3-O-dicaffeoylquinic acid, 3,5-O-caffeoylquinic acid, and 3,4-O-caffeoylquinic acid [7]. Variations in these structures are associated with distinct bioactivities and different therapeutic profiles, and these structural features enable CGA to exert its biological effects on neuropsychiatric disorder models.

### 2.2 Source and Bioavailability

CGAs primarily originate from plants that are widely distributed in daily sources [11]. Generally speaking, CGA is usually consumed through tea, coffee, cocoa, apples, honeysuckle, berries, and pears. It is formed as a secondary metabolite in numerous plant species, particularly in fruits and leaves. The broad presence and biological activities of CGA have made it a frequent subject of research [12].

Despite the promising prospect, its limited oral bioavailability constrains clinical application. Following ingestion, only about one-third of CGAs is absorbed in the small intestine. High-performance liquid chromatography analyses indicate that the main circulating forms are 3-CQA, 4-CQA, and 5-CQA [8]. The remaining fraction enters the large intestine, where microbial metabolism further completes the digestive process. During this phase, the quinic acid component is cleaved, producing ferulic acid and caffeic acid. The latter is subsequently converted into derivatives that can be absorbed in the colon [13].

Although several studies have characterized these metabolic steps, how CGA is absorbed, distributed, utilized, and ultimately eliminated remains unknown. Understanding this gap represents an effective method to translate CGA into therapeutic strategies. Future work should explore more approaches to extend beyond conventional oral administration, including alternative delivery systems and drug combination strategies. Consequently, the therapeutic value of CGA depends on combating these bioavailability limitations and resolving the metabolic limitations.

### 2.3 Pharmacological Effects

Accumulating evidence suggests that dietary CGA may contribute to a lower incidence of many diseases. It possesses numerous pharmacological effects, including anti-cancer effects [14], antioxidant and anti-inflammatory effects [15, 16], antidiabetic [17] and anti-obesity properties [18] and antibacterial effects [19]. CGA has been commonly utilized across the food, health care, pharmaceutical, and chemical industries [20-23].

#### 2.3.1 Anti-cancer Effects of CGA

Recent studies have revealed that CGA can exhibit anticancer effects by repressing the cell cycle, inducing apoptosis, and inhibiting the growth of cancer cells. Researchers demonstrated that CGA could suppress the epithelial-mesenchymal transition and invasion of breast cancer cells by reducing low-density lipoprotein receptor-related protein 6 expression [24]. CGA is a suitable therapeutic method for colon cancer [25]. In Human Tumor-29 (HT-29) cells, CGA triggered cell cycle arrest by enhancing P21 and P53 levels. P21, which is a cyclin-dependent kinase inhibitor, functions as a checkpoint regulator by inhibiting cyclin-CDK complexes, thus halting the cell cycle in response to DNA damage or stress signals. P53, a tumor suppressor protein often referred to as the guardian of the genome, is responsible for activating apoptosis, cell cycle arrest, or DNA repair when the cell experiences severe stress or genetic mutations. Both of these proteins, by enhancing their levels, CGA effectively promotes the prevention of cancer cell proliferation and induces growth inhibition. Another study found that a combination of coffee compounds, particularly those containing CGA, reduces early-stage colon carcinogenesis by modulating the expression of miR-21a-5p. This combination was most effective in reducing epithelial cell proliferation, as measured by Ki-67, and increasing apoptosis, indicated by cleaved caspase-3, in colonic crypts. Additionally, this treatment lowered levels of proinflammatory cytokines while downregulating the oncogenic miR-21a-5p in the colon. Analysis of the targets of miR-21a-5p showed that the genes were involved in inhibiting cell proliferation and inflammation, as well as promoting apoptosis [26]. A study highlighted the potential of CGA to influence the biological mechanisms involved in the development of colorectal cancer, with effects varying according to the treatment doses used [27]. It also promoted apoptosis in HT-29 cells by reducing B-cell lymphoma 2(Bcl-2) and Nuclear Factor kappa B(NF-κB) expression and simultaneously increasing caspase 3, 9, and Reactive Oxygen Species (ROS) levels. In A549 human lung cancer cells, CGA has also been identified to regulate apoptosis and genes associated with stem cell markers [28]. CGA holds potential as a therapeutic agent for renal cell carcinoma [29]. CGA inhibited proliferation and led to apoptosis of A498 human kidney cancer cells *via* deactivating the Phosphoinositide 3-Kinase (PI3K)/Protein Kinase B(Akt)/ Mechanistic Target of Rapamycin(mTOR) signalling pathway. A synergistic effect was observed when CGA was combined with other natural compounds, which were administered to mice implanted with human renal cancer cells [30]. Additionally, CGA inhibited renal cancer cells' growth and angiogenesis.

#### 2.3.2 Antioxidant and Anti-inflammatory Effects of CGA

CGA is renowned for its antioxidant and anti-inflammatory properties. CGA mitigated lipopolysaccharide-induced oxidative stress and inflammation by regulating the CD36/AMP-Activated Protein Kinase (AMPK)/Peroxisome Proliferator-Activated Receptor Gamma Coactivator 1 Alpha (PGC-1α) pathway in RAW264.7 macrophages [31]. CGA mitigated kidney fibrosis *via* modulating TLR4/NF-κB-mediated inflammation and oxidative stress [32]. In the unilateral ureteral obstruction models, CGA substantially alleviated kidney damage, oxidative stress, inflammation, and fibrosis. CGA also effectively suppressed inflammatory factors and the course of oxidative stress, both in fibrosis models. CGA also substantially inhibited inflammatory markers and the oxidative stress pathway expression in fibrosis models, both *in vivo* and *in vitro*. It suppressed the overexpression of lysine acetyltransferase 2A and thus mitigated the inflammation [33]. Furthermore, CGA demonstrated anti-inflammatory effects in a murine acute pancreatitis model [34].

#### 2.3.3 Antidiabetic and Anti-obesity Properties of CGA

The ability of CGA to combat obesity and diabetes is associated with its impact on glucose metabolism. CGA improved microbiota composition, lipid metabolism, glucose tolerance, and inflammation in diabetic leptin receptor-deficient mice [35]. Specifically, it mitigated hepatic lipid levels, normalized inflammatory gene expression such as Tumor Necrosis Factor α (TNF-α), Interleukin 1 beta (IL-1β), and Interleukin 6 (IL-6), Interleukin 10, as well as genes encoding antioxidant enzymes, including Tumor Necrosis Factor 1, and SOD2. Furthermore, CGA enhanced bacterial alpha and beta diversity in the cecum. Notably, it also restored the genera *Lactobacillus*, *Blautia,* and *Enterococcus* and the phylum Bacteroidetes abundance [36]. A study discovered that dietary CGA mitigates high-fat diet (HFD)-induced steatotic liver disease by influencing metabolites and the gut microbiome [37]. To be specific, supplementation with CGA resulted in reduced levels of various lipids, along with elevated levels of l-phenylalanine and l-tryptophan, which promoted the breakdown of fatty acids. CGA treatment was also associated with an enriched abundance of *Muribaculaceae, Bacteroides*, and *Parabacteroides* families. In an HFD-induced prediabetic mouse model, CGA supplementation led to liver lipid metabolism recovery [38]. Consistently, complexes containing CGA can influence glucose homeostasis. Researchers revealed this alternation is realized possibly through interactions with the gut microbiota in type 2 diabetic(T2D) rat models [39]. In another experiment combining HFD with streptozotocin in T2D mice, scientists demonstrated that using a plant rich in CGA could significantly repress islet cell apoptosis and improve pancreatic function [40].

#### 2.3.4 Antibacterial Effects of CGA

Long-term application of antibiotics can lead to drug resistance [41, 42]. Fortunately, CGA can function as a prospective antibacterial agent. It performs antibacterial effects in several mechanisms, including ameliorating ribosomal subunit expression, intervening with lipid metabolism, and reducing intracellular ROS. CGA could prevent *Klebsiella pneumoniae* biofilm formation [43]. Gene expression, such as luxS, wbbm, and mrkA, involved in biofilm formation, is mitigated following treatment with CGA. CGA could also exert its antibacterial effects *via* anti-Inflammatory effects on periodontal tissue to inhibit bacterial proliferation of *Streptococcubacter mutans, actinomycetemcomitans*, and *Fuscobacterium nucleatum* [44]. CGA exhibited significant antibacterial efficacy against *Yersinia enterocolitica* (*Y. enterocolitica*) [19]. CGA could substantially repress biofilm formation and reduce the biomass of established biofilm in *Y. enterocolitica*. CGA potentially disrupted the cell membrane by binding to *Y. enterocolitica*, thus enhancing membrane permeability and leading to damage to the bacterial cell. In addition, CGA also interfered with the antagonizing biofilm process and enhanced cell membrane permeability. CGA could inactivate *Salmonella* *typhimurium* SL1344 and cause damage to its cellular integrity [45].

## 3 EFFICACY OF CGAS IN NEUROPSYCHIATRIC DISORDERS

Accumulating animal experimental evidences suggest that CGA exerts its neuroprotective effects through multiple mechanisms, such as inhibiting false protein aggregation and modulating intracellular pathways associated with neuronal cell survival and death.

Based on these findings, we summarize the major neuroprotective actions of CGA observed across diverse experimental models, with particular emphasis on signaling networks. For more detailed information, please refer to Table **1**. This table provides a representative overview of CGA in experimental animals with neuropsychiatric diseases. It details the dosage of CGA administration, involved signaling pathways, and principal findings. Notably, these findings consistently point to the involvement of inflammatory mediators, oxidative stress pathways, and mitochondrial dysfunction, which together form a shared pathological axis in neurodegenerative conditions (Table **1**).

### 3.1 Parkinson’s Disease

PD is characterized by the degeneration of dopaminergic neurons, intracellular accumulation of α-synuclein, and progressive motor impairment [46]. Traditional treatment commonly relies on dopaminergic replacement strategies, including dopamine receptor agonists, catechol O-methyltransferase inhibitors, and levodopa [47]. Unfortunately, these therapies mainly enhance intracellular dopamine mediator levels and alleviate symptoms only in the early stages; their long-term capacity to alter disease progression remains limited. Therefore, this gap prompted increasing interest in further investigating preventive or disease-modifying therapeutic agents.

#### 3.1.1 Pathological Insights of CGA in PD

Epidemiological evidence suggests that patients who regularly consume coffee tend to reduce PD progression [48, 49]. Although coffee contains a complex mixture of bioactive molecules, CGA has emerged as a candidate for this association. Numerous studies have reported that CGA can attenuate oxidative stress and interfere with pathological α-synuclein(α-syn) aggregation, both of which are closely linked to dopaminergic neurodegeneration [50]. More importantly, CGA has been reported to reduce oxidative stress through free radical scavenging activity, suggesting its potential in PD development [51]. Despite these potential findings, research investigating its effects on PD is still in the early stage, and the precise mechanisms underlying its neuroprotective effects are yet to be fully elucidated.

#### 3.1.2 Evidence of CGA-mediated Neuroprotection in PD Model

The PD-related studies discussed correspond to the Parkinson’s disease entries summarized in Table **1**, including CGA dosage, experimental models, and major molecular pathways (Table **1**).

CGA delayed the PD progression *via* autophagy induction in *Caenorhabditis elegans* (*C. elegans*) [52]. CGA markedly reduced α-syn aggregation, ROS, and malondialdehyde contents in PD *C. elegans*, restored motor disorders, and lipid content. CGA also inhibited dopamine (DA) neuron degeneration and changed food-sensing behavior. Meanwhile, CGA enhanced autophagy-related gene expression, and the anti-PD effect of CGA was closely related to autophagy induction *via* increasing the expression of autophagy-related genes. CGA relieved 1-Methyl-4-phenyl-1,2,3,6-tetrahydropyridine (MPTP)-induced PD-like symptoms, including DA neurons and blood vessel loss, locomotion reduction, and apoptosis events in the brain. Moreover, CGA modulated the expression of PD- and autophagy-related genes, showing its ability to promote autophagy, which was interrupted in the PD pathology [50]. CGA might relieve PD by boosting autophagy in neuronal cells, which makes CGA a potential candidate for anti-PD treatment. Numerous studies suggest that a glucagon-like peptide-1 (GLP-1) secretagogue has a neuroprotective role in PD models. CGA has a strong binding affinity for G-Protein Coupled Receptor-40(GPR-40)/G-Protein Coupled Receptor-43(GPR-43) in comparison to other bioactive polyphenols. In PD mouse models, Sharma N *et al*. demonstrated that CGA significantly enhanced the expression of GLP-1, GPR-40, and GPR-43 mRNA. Furthermore, CGA also mitigated motor and cognitive deficits. This treatment also alleviated rotenone-induced degeneration of dopaminergic neurons and accumulation of α-syn in the substantia nigra, and augmented the density of dopaminergic nerve fibers in the striatum [53]. These results highlight the novel role of CGA as a GLP-1 secretagogue. The upregulation of endogenous GLP-1 by CGA may render neuroprotection in PD mice and holds promise as a potential therapeutic target for managing PD. He *et al*. revealed that CGA inhibited the Extracellular Signal-Regulated Kinase (ERK)1/2 activation and Akt inactivation by eliminating excess ROS in PD models [54]. Another study revealed Eucommia ulmoides Oliv. leaves, rich in CGA, could alleviate PD symptoms. To be specific, it alleviated locomotor impairments, increased the length of dopaminergic neurons, inhibited the loss of neuronal vasculature, and regulated the misexpression of autophagy-related genes [55]. Overall, CGA supplementation could potentially treat PD by alleviating the neuroinflammation and oxidative stress in MPTP-intoxicated mice. CGA served as a potent neuroprotective agent, reducing oxidative stress and endoplasmic reticulum stress in PD [56]. Administration of CGA could also significantly delay the occurrence of PD-like behavioral deficits in rats. CGA also attenuated the activities of antioxidant enzymes and the increased apoptosis in the striatum of rats. CGA may exhibit pharmacological effects and provide neuroprotection against MPTP-induced toxicity in PD mouse models. This protection is close to the phosphorylation of Glycogen Synthase Kinase 3 Beta (GSK3β) through activating the Akt/ERK signaling pathway in the mitochondrial intrinsic apoptotic [57].

Current experiments suggest that CGA exerts neuroprotective actions in PD through coordinated regulation of autophagy, oxidative stress, inflammatory signaling, metabolic pathways, and mitochondrial stability. These joint effects support CGA as a promising treatment for the mitochondrial-mediated apoptotic senescence of DA neurons in PD.

### 3.2 Alzheimer's Disease

AD is a neurodegenerative disease with special characteristics such as dementia, memory impairment, and mental stress. The number of individuals affected by AD and related dementias rose to 51.62 million in 2019 and is predicted to reach 152.8 million by 2050 [58], gaining global attention. Hitherto, the United States Food and Drug Administration has approved only a small number of drugs treating AD [59].

However, the majority of these medications primarily concentrate on alleviating symptoms rather than preventing AD progression.

#### 3.2.1 Pathological Insights of CGA in AD

CGA has exhibited remarkable neuroprotective effects in AD [60]. Evidence from AD animal models suggests that CGA exerts multiple effects, including modulation of autophagic activity and restoration of spatial learning memory. Mechanistically, CGA is frequently reported to restrain NF-κB signaling activation, which is associated with reduced neuroinflammatory toxicity and delayed PD pathological progression [61-63]. Additionally, CGA efficiently fights these AD by activating the PGC1𝛼 and Sirtuin 1(SIRT1) pathway, enhancing mitochondrial efficiency and function, while also exerting antioxidant effects that reduce ROS production [64-66]. CGA features the preventive effects on AD cognitive dysfunction in Amyloid Precursor Protein/Presenilin 1(APP/PS1) transgenic mice. However, the concrete mechanisms of CGA on cognitive damage remain unclear.

#### 3.2.2 Evidence of CGA-mediated Neuroprotection in the AD Model

The findings described below are supported by the AD-specific studies listed in Table **1**, which outline the experimental settings and key mechanistic outcomes of CGA in AD models (Table **1**).

CGA could enhance cognitive performance in mice by alleviating neuroinflammation, oxidative stress, and beta-amyloid (Aβ) deposition. Moreover, when CGA was combined with aerobic exercise, it would exert neuroprotective effects and thus reverse cognitive decline in the APP/PS1 transgenic mouse model through activating the SIRT1/PGC-1α/PPARγ signaling pathway [67]. Bezerra JR *et al*. revealed the neuroprotective properties of CGA in an animal model of sporadic AD induced by streptozotocin [68]. The CGA treatment ameliorated spatial memory, alleviating neuronal damage and repressing autophagy in APP/PS1 mice. Furthermore, in SH-SY5Y cells stimulated by Aβ25-35, CGA effectively reduced the formation of autophagosomes and boosted autophagy flux. CGA also significantly promoted lysosomal activity, which was reflected in the increased cathepsin D protein expression [69]. CGA can protect early AD rats from cognitive impairment. It increased Nissl level, downgraded inflammatory factors expression, reduced GSK3β, GFAP, and tau expression, and enhanced dishevelled segment polarity protein 2 and β-catenin expression [70]. A study revealed that CGA could modulate some of the therapeutic properties of donepezil. Specifically, CGA had a substantial impact on how donepezil alleviated elevated butyrylcholinesterase activity in rats that received CGA [71]. However, when CGA was administered orally at a low dose, its ability to suppress Aβ deposition and neuroinflammatory responses in the brain was impaired, and glucose homeostasis disorders still exist. Furthermore, it demonstrated limited efficacy in restoring microbial composition. Li *et al*. engineered a brain-targeted selenium nanocluster that enhanced the delivery efficiency of CGA, allowing a significant reduction in the dose application and leading to measurable cognitive improvement in APP/PS1 mice [72].

Collectively, although current findings revealed a neuroprotective role for CGA in AD-related models, the specific mechanisms remain uncertain. Further investigation is required to determine whether these effects can be utilized in clinical applications.

### 3.3 Stroke

Stroke is a major contributor to disability and mortality worldwide. Among its subtypes, ischemic stroke accounts for approximately 80-85% of recent cases. Ischemic stroke arises from blocked cerebral perfusion, which deprives local tissue of oxygen and metabolic activity. In addition, accumulating evidence indicates that excessive oxidative stress is a driving factor in the cascade of neuronal injury following ischemia [73].

#### 3.3.1 Pathological Insights of CGA in Stroke

In mice with focal cerebral ischemia, CGA administration was related to reduced brain edema and infarct volume, with improved neurological performance. These effects were accompanied by decreased ROS levels and enhanced antioxidant enzyme activity, including catalase and SOD [74, 75]. Therefore, CGA appears to influence several components of the ischemic injury cascade, contributing to the preservation of neuronal viability during the acute phase of stroke. Moreover, it has been found to improve behaviors, suggesting its potential in neurodegenerative disorders related to oxidative damage. Given its diverse anti-oxidative effects, CGA offers therapeutic potential in stroke management, highlighting its role in future clinical applications for enhancing brain recovery and reducing oxidative damage.

#### 3.3.2 Evidence of CGA-mediated Neuroprotection in Stroke Model

Table **1** describes the effects of CGA in depression at length (Table **1**).

CGA could regulate proteins in the ubiquitin-proteasome system and safeguard cortical neurons from damage caused by ischemic brain injury [76]. In a separate study concerning the MCAO-induced brain damage rat model, the animals exhibited neurological impairments, alleviated thioredoxin expression, and enhanced levels of ROS and lipid peroxidation. Luckily, these changes were reversed by CGA treatment. Furthermore, the interaction between thioredoxin and apoptosis signal-regulating kinase 1 was diminished in MCAO rats, and CGA treatment effectively reversed this reduction [77]. Namely, CGA exerted a neuroprotective efficacy by modulating thioredoxin expression under cerebral ischemia and glutamate exposure conditions. Shah MA *et al*. demonstrated that CGA could provide neuroprotection by activating the Akt-Bad signaling pathway and preserving the interaction between phospho-Bad and 14-3-3 protein in ischemic stroke models [78]. CGA also demonstrated neuroprotective effects by modulating oxidative stress and the expression of caspase proteins in MCAO injury [79]. Kang *et al*. discovered that CGA modulated the protein phosphatase 2A subunit B expression in the cerebral cortex of a rat stroke model and in neurons which exposed to glutamate [80]. CGA prominently mitigated apoptotic damage and twisted molecular alterations in cerebral cortex neurons after ischemia-reperfusion (IR) [81]. The miR-27a/smurf1/TNF-α axis played a regulatory function in cerebral cortex cell death, potentially providing a new therapeutic target for transient ischemic stroke. A study noted enhanced miR-27a gene expression, TNF-α protein level, as well as reduced Smurf1 protein expression following IR. Notably, CGA treatment could restore these molecular changes. CGA protected neurons by reducing oxidative stress and regulating pro-inflammatory proteins in cerebral ischemic injury [82]. It demonstrates antioxidant effects and a clear anti-inflammatory mechanism by regulating IL-1β, NF-κB, and TNF-α in cerebral ischemia. CGA mitigated the neurological impairments caused by MCAO [83]. Additionally, it modulated the expression of proteins related to neuroprotection. CGA treatment provided neuroprotection towards global ischemic injury by repressing various molecular markers concerning cerebral ischemia [84]. Specifically, when compared with ischemic controls, CGA treatment was related to reduced cerebral infarction areas, with a decrease in calcium, nitrate, and glutamate levels across several brain regions, including the cortex, cerebellum, hippocampus, and cerebrospinal fluid. In parallel, TNF-α, inducible nitric oxide synthase (iNOS), and caspase-3 levels were observed to reduce after CGA administration. Usually, the miR-23b/TAB3/NF-κB/p53 signaling axis could exert regulatory effects on hippocampal cell death. A research study found that CGA could reverse these molecular changes. CGA could alleviate hippocampus apoptosis following acute IR injury [85].

CGA can exert protective effects in stroke through several molecular routes. For instance, it modulates proteins in the ubiquitin-proteasome system, influencing oxidative stress levels and pro-inflammatory proteins. These mechanisms work together to help to minimize the harm to neurons. Taken together, these findings imply that CGA acts on multiple regulatory levels in the treatment of stroke, although its translational value in clinical settings remains to be established.

### 3.4 Depression

Depression affects a substantial proportion of the global population, with approximately 350 million individuals worldwide [86]. As a major factor for depression, chronic stress is commonly associated with persistent low mood, anhedonia, and impaired social activity. Despite extensive research efforts, the biological mechanisms underlying depression remain unknown. Conventional clinical treatments fail to produce satisfactory effects in a considerable proportion of patients. Around two-thirds of patients experienced adverse effects and still require prolonged treatment, while a significant subgroup shows little or no response to diverse pharmacological interventions [87]. These limitations highlight the continuous need for deeper investigation into the pathophysiological basis of depression.

#### 3.4.1 Pathological Insights of CGA in Depression

Recent studies suggest that CGA may exert neuroprotective effects in the depression process. Experimental evidence indicated that these effects are associated with multiple biological processes, including the regulation of oxidative stress, modulation in gut microbiota composition, and changes in metabolic activity [88, 89]. CGA treatment has been linked to improvements in both motor function and cognitive performance. Furthermore, several studies have reported beneficial effects of CGA on anxiety-like behaviour, depressive symptoms, and learning-related deficits [90]. CGA has been shown to alleviate depression-like phenotypes in experimental models. In parallel, accumulating studies report a reverse association between habitual coffee intake and depressive symptoms, indirectly supporting a role for CGA-related dietary exposure in mood regulation [91]. These findings suggest that CGA acts as a therapeutic agent for treating depression. Nonetheless, more research is required to explore its clinical potential.

#### 3.4.2 Evidence of CGA-mediated Neuroprotection in the Depression Model

A detailed overview of the animal studies cited here, including behavioral outcomes and molecular pathways, is provided in the depression section of Table **1** (Table **1**).

CGA is a crucial constituent of *Lonicera japonica* flos (LJF). Both the extracts of LJF and CGA exhibited substantial antidepressant efficacy. In fact, the antidepressant efficacy of various LJF extracts is positively correlated with their CGA content. Furthermore, the 70% ethanolic extract of LJF and CGA ameliorated depressive behaviors. They also increased the protein expression levels of Brain-Derived Neurotrophic Factor (BDNF) and phosphorylated TrkB (p-TrkB) in the hippocampus, and helped to mend damage to hippocampal neurons [92]. CGA could alter the composition of the gut microbiota and thus improve depression-like behaviors in rats [93]. Lim *et al*. discovered that the extract from dried loquat fruit containing CGA could repress corticosteroid-induced depressive-like behaviors [90]. In another experiment, a researcher showed that CGA derived from hawthorn berry could inhibit depressive behavior. This result was mediated *via* the monoamine oxidase B - reactive oxygen species signaling pathway in the hippocampal astrocytes of mice [94]. In SD rat models, CGA demonstrated significant antidepressant effects, indicating its mechanism may involve the modulation of several metabolic pathways, including nicotinamide and nicotinate metabolism, dicarboxylate and glyoxylate metabolism, serine, glycine, and threonine metabolism, as well as arginine and proline metabolism [95]. Another research also revealed that CGA could impact metabolic pathways to alleviate depressive behaviors [96].

These findings indicate that CGA may influence depression-related behaviors *via* a combination of central neurotrophic signaling and peripheral metabolic pathways. To be specific, CGA administration was associated with elevated BDNF and p-TrkB protein levels, with structural improvements in neuronal integrity and gut microbiota alterations in hippocampal tissue. Beyond central mechanisms, CGA also attenuated corticosteroid-induced depression, possibly through interactions with gut microbial profiles and downstream metabolic regulation.

### 3.5 Epilepsy

Despite substantial advances in clinical management, epilepsy continues to impose a considerable burden on patients. A substantial number of patients still fail to achieve seizure remission under existing therapeutic regimens [97]. This limitation stimulates the need to reassess current treatment approaches and investigate novel intervention strategies.

#### 3.5.1 Pathological Insights of CGA in Epilepsy

CGA has been reported to penetrate the blood-brain barrier and attenuate neuronal injury in status epilepticus (SE) models, particularly following oral administration. Much evidence suggests that CGA could exert its neuroprotective effects against epilepsy, which are associated with the modulation of excitatory and inhibitory neurotransmission, together with a reduction in oxidative stress and inflammatory responses [98-100].

Excessive generation of free radicals during status epilepticus has been implicated in glutamate-driven neuronal damage and is closely linked to apoptotic neurodegeneration. Evidence from experimental studies suggests that CGA may confer neuroprotection in epilepsy, at least in part by limiting oxidative stress and inflammatory signaling. These properties may help in the development of complementary therapeutic approaches for treating epilepsy and attenuating its long-term neurotoxic effects.

#### 3.5.2 Evidence of CGA-mediated Neuroprotection in the Depression Model

 The epilepsy-related effects of CGA described above are supported by the studies in Table **1**, which encompass seizure animal models and examine associated changes in cytokine profiles and neurotransmitter-related mechanisms (Table **1**).

In a comparative experiment, CGA could mitigate the epilepsy behavioral alterations [100]. CGA administration was associated with marked increases in brain-derived neurotrophic factor levels and acetylcholinesterase activity, together with elevated concentrations of monoamine neurotransmitters, including dopamine, norepinephrine, and serotonin. In parallel, CGA enhanced endogenous antioxidant capacity in mice. Treatment also resulted in a clear reduction in pro-inflammatory signalling, as reflected by lower expression of IL-6, TNF-α, IL-1β, NF-κB, and inducible nitric oxide synthase in epileptic animals. At the cellular level, CGA was found to mitigate neuronal apoptosis in the hippocampus, as reflected by reduced caspase-3 and Bax expression together with a recovery of Bcl-2 levels. A study demonstrated that CGA can block glutamate receptors [101]. This study proposed that CGA could ameliorate symptoms of epilepsy, which was closely related to glutamatergic signaling modulation. In this context, CGA appeared to suppress the activity of N-methyl-D-aspartate receptors as well as metabotropic glutamate receptors 1 and 5, influencing calcium-dependent neuronal vulnerability. Carreño-González *et al*. reported that CGA administration at 30 mg/kg significantly ameliorated the symptoms of epilepsy by reducing oxidative stress. This protective effect persisted until the peak of neural damage, occurring 72 hours later [102]. The daily administration of CGA significantly mitigated epilepsy-like seizures and cognitive deficits, improving hippocampal neuroinflammation and neuronal degeneration. CGA exerted neuroprotective properties *via* the c-Jun N-terminal Kinase/ c-Jun and TLR4/NF-κB axis. In epilepsy mice, CGA enhanced the abundance of Lactobacillus [98]. Moreover, neuroprotective gut microbial metabolites, such as short-chain fatty acids, increased in the mouse hippocampus. Oral gavage of CGA also regulated neurotransmitter imbalances.

In summary, these findings indicate that CGA could mediate the neuroprotective efficacy against epilepsy *via* regulation of neurotransmitters, neuroinflammation, oxidative damage, and apoptosis. CGA, therefore, could probably be a valuable therapeutic supplement for epilepsy treatment.

### 3.6 Other Neuropsychiatric Disorders

Additional CGA studies relevant to anxiety-like behaviors, PTSD, and toxin-induced neurotoxicity are also catalogued in Table **1** (Table **1**).

CGA, particularly combined with syringaresinol-di-O-β-D-glucoside, has been implicated in reducing anxious phenotypes and autonomic regulation. It was also found to elevate hippocampal BDNF signaling and ultimately suppress pharmacological anxiety-related behaviors [103].

Post-traumatic stress disorder (PTSD) is a chronic, delayed psychiatric syndrome induced by sudden, threatening, or catastrophic experiences. One study demonstrated that CGA mitigated single-prolonged-stress-induced PTSD-like manifestations by repressing hippocampal neuronal apoptosis, inhibiting neuroinflammatory and oxidative insult, and enhancing synaptic plasticity *via* nuclear factor erythroid 2-related factor 2/NF-κB signaling axis [104].

Systemic exposure to 3-nitropropionic acid (3-NP) damaged striatal medium spiny neurons of the basal ganglia, mirroring Huntington’s disease pathology. Combination 3-NP with CGA markedly reduced cerebral lipid peroxidation and fostered an anti-inflammatory profile in the brain [75]. Collectively, this finding supported that CGA may function as a broad-spectrum neuroprotective agent against environmental toxins that impair mitochondrial integrity.

Schizophrenia can be effectively treated with Olanzapine. However, this drug usually brings many side effects, especially accompanied by metabolic disturbances. Fortunately, CGA has been reported to attenuate these adverse effects through multiple mechanisms, including modulating leptin, neuropeptide Y, pro-opiomelanocortin, and AMPK signaling [105]. Therefore, CGA can potentially be a dietary remedy in alleviating antipsychotic-associated metabolic burden.

At present, evidence supporting CGA in neuropsychiatric disorders remains largely limited. And this evidence is predominantly derived from animal experiments. Further investigations are required to clarify its efficacy and translational value in relevant clinical applications.

## 4 FUTURE AND PERSPECTIVES

CGA is among the most extensively investigated dietary polyphenols. Many researchers have been increasingly exploring its potential relevance in neuropsychiatric research. Despite growing experimental work supporting that CGA may engage many molecular pathways, which are distinct from those targeted by other polyphenolic compounds, its clinical development remains limited. One important constraint is its poor oral bioavailability, which blocks its therapeutic concentration *in vivo* [106]. Fortunately, this pharmacokinetic limitation prompted research in delivery systems, including nano-scale carriers and other strategies aimed at improving central nervous system exposure. In parallel, several studies have proposed that combined medications can amplify the efficacy of CGA treatment, particularly in complex and heterogeneous pathophysiology disorders [72, 107, 108]. While such combination approaches are seemingly attractive, translational application remains uncertain, as most of which is derived from preclinical models. Therefore, rigorous clinical trials are required to determine whether combined medications of CGA can offer benefits in humans. Notably, some problems, including dose-response relationships, safety profiles, and drug combinations, should be properly handled under any circumstances [109-111]. Beyond translational considerations, the related mechanism still remains unknown. Although the efficacy of CGA is closely related to its antioxidant efficacy and neurotransmitter signaling, it is unclear whether these effects reflect a unified molecular programme or mechanistically distinct processes. Understanding how CGA interacts with molecular targets, together with genetic and epigenetic responses to CGA exposure, is essential for determining whether CGA can be applied in neuropsychiatric disorders clinically.

## CONCLUSION

CGA has been increasingly discussed in experimental models of neuropsychiatric disorders for its superb neuroprotective ability. Current evidences suggest that its effects emerge from multiple biological processes, including inflammatory signaling, redox regulation, autophagic flux, and the handling of misfolded or aggregated proteins. Some studies also point out that CGA can preserve synaptic structure and function, implying that its influence extends beyond cellular survival to maintain neural network stability.

Fig. (**2**) provides a conclusion of major mechanisms mediated by CGA in neuropsychiatric disorders. (Fig. **2**) Oxidative stress conditions can enhance SOD activity and activate the Nrf2/HO-1 axis. In parallel, CGA can attenuate neuroinflammatory responses through reducing pro-inflammatory cytokine release, including TNF-α and IL-1β. CGA also influences autophagic processes, which may facilitate the clearance of dysfunctional proteins and organelles. In addition, CGA has been associated with the modulation of neurotrophic signaling, such as the BDNF/TrkB and SIRT1/PGC-1 pathways, suggesting a potential link between its metabolic effects and synaptic maintenance.

Although limited bioavailability remains a major obstacle for clinical translation, the consistency of preclinical findings indicates that CGA represents a neuroprotective compound. Further works are required to enhance its pharmacokinetic efficiency and to determine whether its multi-target profile can be applied in clinical settings.

## Figures and Tables

**Fig. (1):**
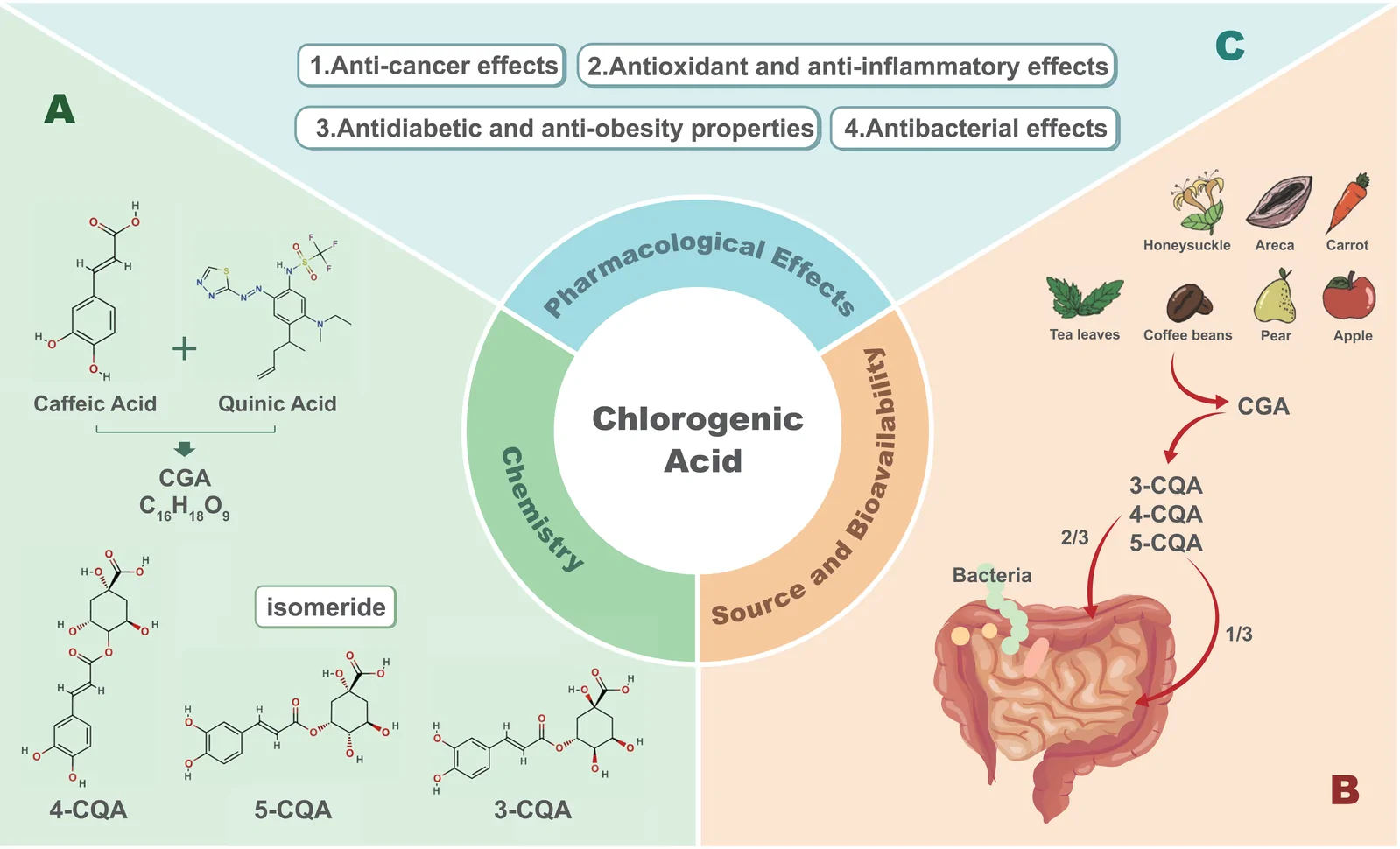
The characteristics of chlorogenic acid in (**A**) Chemistry, (**B**) Source, bioavailability, and (**C**) Pharmacological effects. Part A showed that CGA is formed *via* esterification of caffeic acid with quinic acid. CGAs have three important existence forms: 3-CQA, 4-CQA, and 5-CQA. Part B demonstrated the source of CGA and its bioavailability of CGA *in vivo*. CGA can be converted into 3-CQA, 4-CQA, and 5-CQA during digestion. In the small intestine, one-third of CGA is absorbed, and the remaining two-thirds of CGA pass into the large intestine, where they are metabolized by the gut microbiota to deepen the bioavailability process. CGA, chlorogenic acid; 3-CQA, 3-caffeoylquinic acid; 4-CQA, cryptochlorogenic acid; 5-CQA, neochlorogenic acid.

**Fig. (2):**
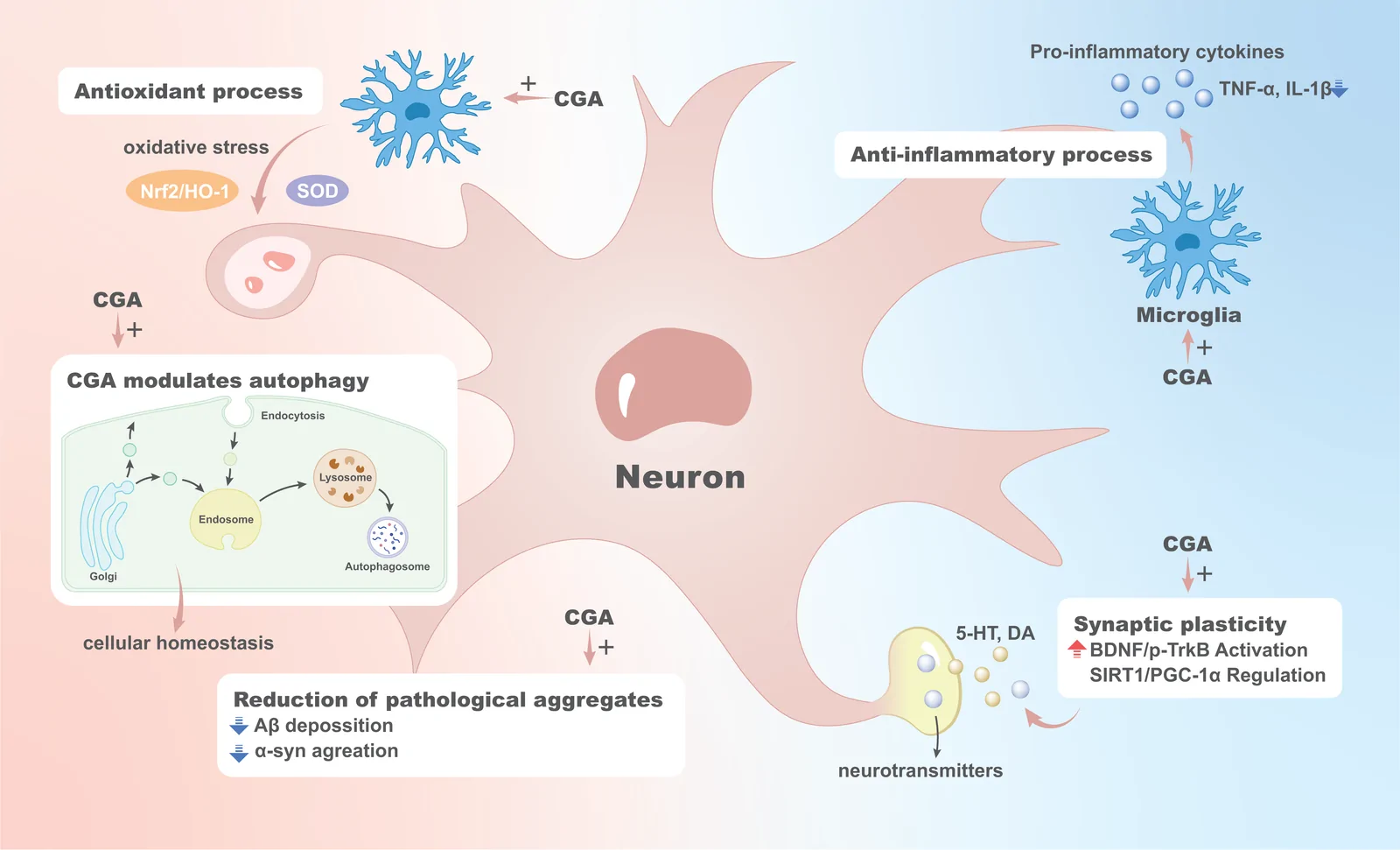
Summary of the overall mechanisms of CGA in neuroprotective effects. This figure illustrates major mechanisms in the neuroprotective effects of CGA. CGA reduces oxidative stress by enhancing SOD levels and activating the Nrf2/HO-1 pathway. It suppresses neuroinflammation by decreasing pro-inflammatory cytokines such as TNF-α and IL-1β production. CGA can also modulate autophagy and thereby contribute to cellular homeostasis. Additionally, CGA promotes neurotrophic signaling such as BDNF/p-TrkB and SIRT1/PGC-1. CGA also improves neurotransmitter balance, supporting synaptic plasticity. Together, these mechanisms converge to maintain neuronal morphology and enhance neural resilience. BDNF, brain-derived neurotrophic factor; CGA, chlorogenic acid; HO-1, heme oxygenase-1; IL-1β, interleukin-1 β; Nrf2, nuclear factor erythroid 2-related factor 2; p-TrkB, phosphorylated TrkB; PGC-1, peroxisome proliferator-activated receptor gamma coactivator 1; SIRT1, sirtuin 1; SOD, superoxide dismutase; TNF-α, tumor necrosis factor α.

**Table 1:** Neuroprotective effects of CGA.

**Diseases**	**Subject**	**Dose**	**Pathways**	**Major Findings**
PD	Caenorhabditis elegans	50-200μM	Autophagy induction	↓α-synuclein aggregation; ROS and MDA contents; DA neuron-degeneration; ↑motor disorders, restored lipid content, food-sensing behavior; autophagy-related genes expression [52]
PD	Wild-type AB zebrafish	Different concentration gradients	Autophagy induction	↓PD-like symptoms; ↑autophagy [50]
PD	C57BL/6 mice	50 mg/kg/d	PI3K/AKT passway	↑GLP-1 and GPR-40 and GPR-43 mRNA expression; dopaminergic nerve fibers mean density; ↓motor and cognitive impairments; dopaminergic nerve degeneration and α-synuclein accumulation [53]
PD	WT mice	50 mg/kg/d	Akt/Erk1/2 passway	↓PD toxins-induced large amount of ROS; Erk1/2 activation; Akt inhibition; neuronal cell death [54].
PD	SD rats	Different concentration gradients	Endoplasmic reticulum stress	↑Apoptosis; ↓oxidative stress; endoplasmic reticulum stress; PD-like behavioral impairments; antioxidant enzymes activity [56]
PD	Swiss albino mice	50 mg/kg/d	Akt/ERK passway	↓the fall in activity of mitochondrial complexes I, IV, and V; superoxide dismutase and mitochondrial glutathione levels; activation of proapoptotic proteins; ↑antiapoptotic protein Bcl-2 [57]
AD	C57BL/6 mice	80 mg/kg/d	SIRT1/PGC-1α/PPARγ pathway	↑oxidative stress, neuroinflammation, and cognitive performance ↓Aβ deposition [67]
AD	Albino Swiss mice	5 mg/kg/d	BDNF	↓BDNF depletion; astrogliosis and microgliosis in the prefrontal cortex and hippocampus [68]
AD	APP/PS1 double transgenic C57BL/6J mice	40 mg/kg/d	mTOR/TFEB pathway	↑Lysosomal Activity in SH-SY5Y Cells; ↓Cognitive Deficits and Neuronal Damage; Autophagy in SH-SY5Y Cells [69]
AD	SD rat	150 mg/kg/d	Wnt pathway	↑Nissl level; DVL2 and β-catenin expression; ↓inflammatory factors expression; GSK-3β, GFAP; tau expression; DVL2 and β-catenin expression [70]
AD	Wistar strain albino rats	8 mg/kg/d	AChE or BChE active sites	↓Elevation in BChE activity [71]
AD	APP/PS1 transgenic mice and wild-type mice	80 mg/kg/d	-	↑Improving brain glucose homeostasis; regulating Aβ aggregation [72]
Stroke	SD rats	30 mg/kg/d	Ubiquitin-proteasome system	Reversed MCAO damage [76]
Stroke	SD rats	30 mg/kg/d	Regulate thioredoxin expression	↑Thioredoxin expression and increased levels; interaction between thioredoxin and ASK1; neurological defects; ROS and lipid peroxidation levels [77]
Stroke	SD rats	30 mg/kg/d	Akt-Bad pathway	↑Phospho-PDK1, phospho-Akt expression; interaction between phospho-Bad and 14-3-3 levels; ↓terminal deoxynucleotidyl transferase dUTP nick end labeling-positive cells; the increase of cytochrome c and caspase-3 expression [78]
Stroke	SD rats	30 mg/kg/d	Apoptotic-related proteins	↓Terminal deoxynucleotidyl transferase dUTP nick end labeling-positive cells; Caspase-3, caspase-7, and poly ADP-ribose polymerase expression levels [79]
Stroke	SD rats	30 mg/ kg/d	Phosphatase 2A subunit B expression and glutamate-exposed	↑PP2A subunit B expression; ↓glutamate-mediated excitotoxicity; Protected neurons [80]
Stroke	Wistar rats	30 mg/kg/d	miR-27a/smurf1/ TNF-α pathway	↓miR-27a gene expression, TNF-α level as ↑ smurf1 protein expression [81]
Stroke	SD rats	30 mg/kg/d	NF-κB pathway	↓NF-κB, IL-1β, TNF-α; microglia and astrocyte activation markers [82]
Stroke	SD rats	30 mg/ kg/d	The expression of proteins involved in neuroprotection	↑Adenosylhomocysteinase, glycerol-3-phosphate dehydrogenase, eukaryotic translation initiation factor 4A-II, apolipoprotein A-I, and mu-crystallin [83]
Stroke	Charles foster albino rats	Different concentration gradients	TNF-α, iNOS, and Caspase-3	↑ Cerebral infarction area; calcium, nitrate, and glutamate levels; expression of TNF-α, iNOS, and caspase-3 [84]
Stroke	Wistar rats	30 mg/kg/d	miR-23b/TAB3/ NF-κB/p53 axis	↑ miR-23b gene expression; ↓apoptotic damage; TAB3 and NF-κB protein expressions [85]
Depression	ICR mice	Different concentration gradients	BDNF-TrkB pathway	↑BDNF and p-TrkB expression; ↓the damage of hippocampal neurons; depressive behavior [92]
Depression	Wistar rats	500 mg/kg	Gut-brain axis	↑5-HT, DA; ↓IL-6, and TNF-α; the decrease in fecal microbiota diversity; depression-like behavior [93]
Depression	ICR mice	30 mg/kg/d	Antioxidant-related mechanisms	↑Cognitive function; ↓depressive-like behaviors; anxiety-like behaviors [90]
Depression	ICR mice	30 mg/kg/d	monoamine oxidase B-reactive oxygen species signaling	↓MAOB activity; ROS production; depressive behaviors [94]
Depression	SD rats	50 mg/kg/day	Metabolic pathways	↓Depressive-like behaviors [95]
Depression	Wistar rats	500 mg/kg	Metabolic pathways	↑5-HT, DA; ↓depressive-like behaviors [96]
Epilepsy	Swiss mice	200 mg/kg/d	antioxidant	↑Bcl-2, ↓iNOS, IL-1β, IL-6, TNF-α, NF-κB, Bax, caspase-3 [100]
Epilepsy	Swiss albino mice	5 mg/ kg/d	NMDA receptors	↓NMDAR, mGluR1 and mGlu5 expression; lipid peroxidation and nitrite content [9]
Epilepsy	Wistar rats	30 mg/kg/d	Oxidative stress	↓Oxidative stress [101]
Epilepsy	C57 BL/6J mice	30 mg/kg/d	JNK/c-Jun and TLR4/NFκB passway	↑Lactobacillus abundance; metabolites associated with neuroprotection; ↓epilepsy-like seizure and cognition impairment; hippocampal neuronal degeneration and neuroinflammation [98]
Anxiety	SD rats	40 mg/kg/d	BDNF-TrkB signaling	↓Anxiety; ↑BDNF [102]
PTSD	SD rats	Different concentration gradients	Nrf2 and NF-κB signaling pathway	↓Hippocampal neuronal apoptosis, neuroinflammatory and oxdiative stress response, PTSD-like behaviors [103]
Neurodegeneration	C57BL/6 mice	100 mg/kg/d	Lipid peroxidation	↑Brain anti-inflammation [75]
Antipsychotic-induced metabolic disturbances	Wistar rats	Different concentration gradients	Hypothalamic satiety proteins	↓Substantial hyperphagia, weight gain, hypertension, liver damage, elevated fasting blood glucose, triglycerides, and leptin levels caused by Olz [105]

## LIST OF ABBREVIATIONS

3-NP: 3-nitropropionic Acid
4-CQA: Cryptochlorogenic Acid
5-CQA: Neochlorogenic Acid
AD: Alzheimer’s Disease
Akt: Protein Kinase B
AMPK: AMP-Activated Protein Kinase
APP/PS1: Amyloid Precursor Protein/Presenilin 1
Aβ: Beta-amyloid
Bcl-2: B-cell Lymphoma 2
BDNF: Brain-Derived Neurotrophic Factor
*C. elegans*: *Caenorhabditis elegans*
CGA: Chlorogenic Acid
CQA: Caffeoyl Quinic Acid
DA: Dopamine
ERK: Extracellular Signal-Regulated Kinase
GFAP: Glial Fibrillary Acidic Protein
GLP-1: Glucagon-like Peptide-1
GPR-40: G-Protein Coupled Receptor-40
GPR-43: G-Protein Coupled Receptor-43
GSK3β: Glycogen Synthase Kinase 3 Beta
HFD: High-fat Diet
HT-29: Human Tumor-29
IL-1β: Interleukin 1 beta
IL-6: Interleukin 6
iNOS: Nitric Oxide Synthase
IR: Ischemia-reperfusion
IUPAC: International Union of Pure and Applied Chemistry
LJF: *Lonicera Japonica* Flos
MCAO: Middle Cerebral Artery Occlusion
mGluR: Metabotropic Glutamate Receptor
MPTP: Methyl-4-phenyl-1,2,3,6-tetrahydropyridine
mTOR: Mechanistic Target of Rapamycin
NF-κB: Nuclear Factor kappa B
NMDAR: N-Methyl-D-Aspartate Receptor
PD: Parkinson’s disease
PGC-1α: Peroxisome Proliferator-Activated Receptor Gamma Coactivator 1 Alpha
PI3K: Phosphoinositide 3-Kinase
PTSD: Post-traumatic Stress Disorder
ROS: Reactive Oxygen Species
SE: Status Epilepticus
SIRT1: Sirtuin 1
T2D: Type 2 diabetic
TNF-α: Tumor Necrosis Factor α
TRKB: p-Tropomyosin Receptor Kinase B
α-syn: α-synuclein
